# Dataset of NMR-spectra pyrrolyl- and indolylazines and evidence of their ability to induce heat shock genes expression in human neurons

**DOI:** 10.1016/j.dib.2021.107562

**Published:** 2021-11-12

**Authors:** Elizaveta A. Dutysheva, Irina A. Utepova, Maria A. Trestsova, Alexander S. Anisimov, Valery N. Charushin, Oleg N. Chupakhin, Boris A. Margulis, Irina V. Guzhova, Vladimir F. Lazarev

**Affiliations:** a Russian Academy of Sciences, Institute of Cytology, St. Petersburg 194064, Russia; b Ural Federal University, Ekaterinburg 620002, Russia; c Postovsky Institute of Organic Synthesis, Ural Branch of the Russian Academy of Sciences, Ekaterinburg 620108, Russia

**Keywords:** Pyrrolylazines, Indolylazines, Photocatalysis, Nuclear magnetic resonance, Green chemistry, Heat shock genes

## Abstract

These data are related to our previous paper “Synthesis and approbation of new neuroprotective chemicals of pyrrolyl- and indolylazine classes in a cell model of Alzheimer's disease” (Dutysheva et al., 2021), in which we demonstrate neuroprotective abilities of pyrrolyl- and indolylazines in a cell model of Alzheimer's disease. Using a novel procedure of photocatalysis we have synthesized a group of new compounds. The current article presents nuclear magnetic resonance spectra including heteronuclear single quantum coherence spectra of chemicals synthesized by us. The effect of new compounds have on heat shock proteins genes expression in reprogrammed human neurons are presented. We also presented data that verify neuronal phenotype of reprogrammed cells.

## Specifications Table

**Table:** 

Subject	Molecular medicine
Specific subject area	Induction of protein synthesis using chemical compounds from the azolazine class.
Type of data	Graph NMR-Spectra
How data were acquired	Nuclear magnetic resonance, real time polymerase chain reaction
Data format	Analysed
Parameters for data collection	^1^H NMR (400 MHz) and ^13^C NMR (100 MHz) spectra were recorded on a Bruker Avance II, using SiMe_4_ as internal reference in DMSO-*d*_6_ and CDCl_3_. Real time polymerase chain reactions were performed with a CFX96 Real-Time PCR detection system (BioRad, USA) using qPCRmix-HS SYBR (Evrogen JSC, Russia) according to the manufacturer's protocol.
Description of data collection	The Nuclear magnetic resonance spectra for pyrrolyl- and indolylazines were registered. The level of gene expression was assigned with the aid of real-time polymerase chain reaction.
Data source location	Institution: Ural Federal University City/Town/Region: Ekaterinburg Country: Russia Institution: Institute of Cytology, Russian Academy of Sciences City/Town/Region: St. Petersburg Country: Russia
Data accessibility	With the article
Related research article	E.A. Dutysheva, I.A. Utepova, M.A. Trestsova, A.S. Anisimov, V.N. Charushin, O.N. Chupachin, B.A. Margulis, I.V. Guzhova, V.F. Lazarev. Synthesis and approbation of new neuroprotective chemicals of pyrrolyl- and indolylazine classes in a cell model of Alzheimer's disease, Eur. J. Med. Chem. 222 (2021) 113,577. DOI: 10.1016/j.ejmech.2021.113577

**Value of the Data**

• The current paper illustrates the nuclear magnetic resonance spectra of newly synthesised chemical compounds of pyrrolyl- and indolyl classes.
• The data presented in this article may be beneficial to the search for new azolylazines and their synthesis for application in the field of neuroprotection.
• A possible application of the presented data lies in the use for prediction of biological activity spectra for substances

## 1 Data Description

Compounds from the pyrrolyl and indolylazine classes have demonstrated their therapeutic potential more than once. Previously, for compounds from these classes it was demonstrated antineoplastic (**THZ531** [1], **Variolin B** [2]), antianginal (**BDBM92080** [3]), cytotoxic (**Meriolin 2** [4], **Hyrtinadine A** [5]), and antiasthmatic (**US9999619** [6]) activities, as well as including α-adrenoceptor blocking agents (**Nicergoline** [7]) and potent inhibitors of serine-threonine protein phosphatases. This work presents the data of nuclear magnetic resonance (NMR) analysis of the pyrrolyl- and indolylazines synthesized by us, which previously demonstrated neuroprotective potential in the cellular model of Alzheimer's disease [8] and in the model of traumatic brain injury [9].

**Fig. 1:**
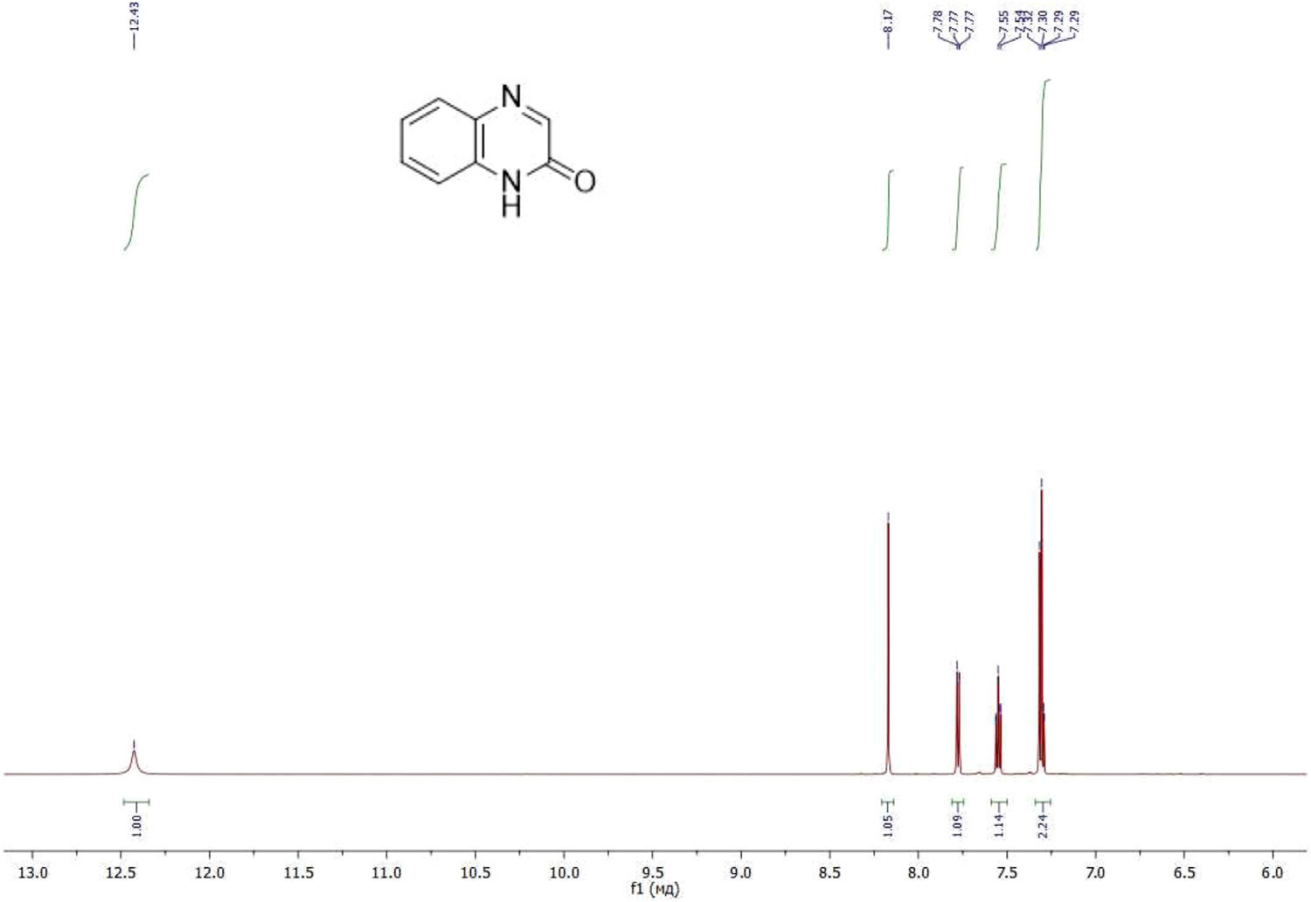
^1^H NMR (400 MHz, DMSO-d6) spectrum for quinoxalin-2(1H)-one **1**.

**Fig. 2:**
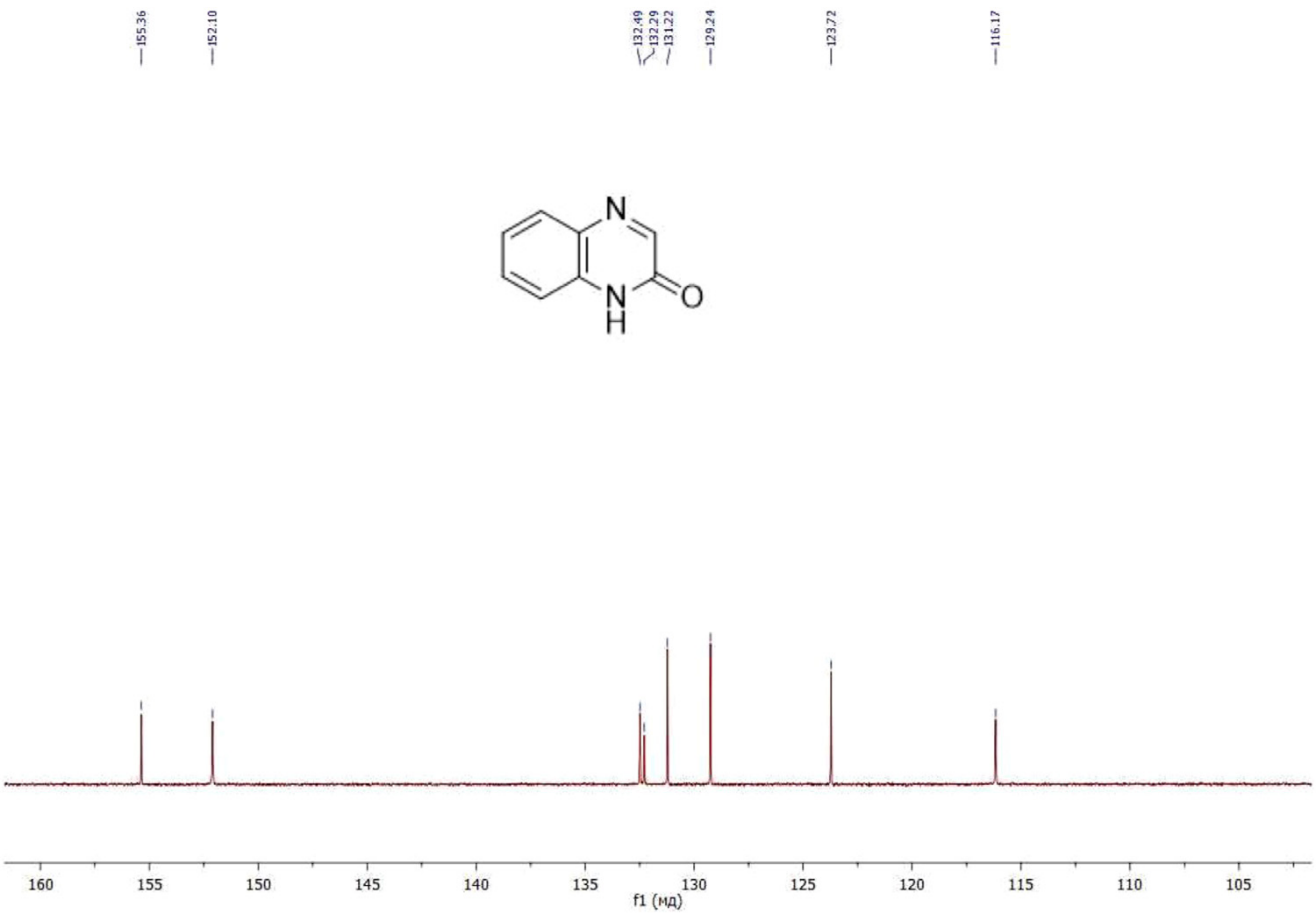
^13^C NMR (100 MHz, DMSO-d6) spectrum for quinoxalin-2(1H)-one **1**.

**Fig. 3:**
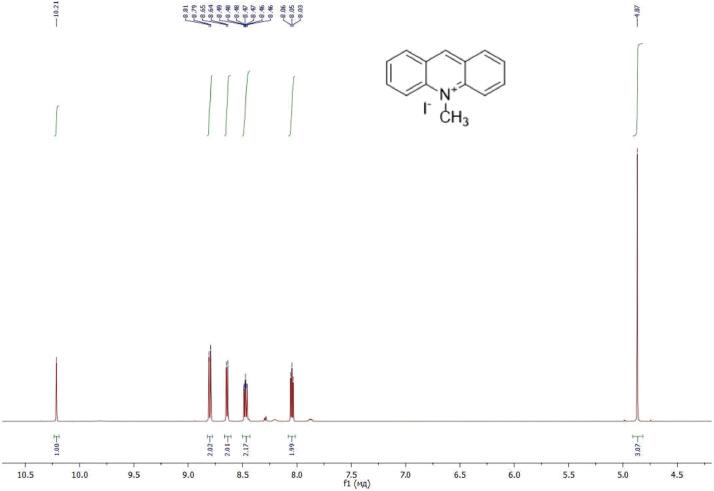
^1^H NMR (400 MHz, DMSO-d6) spectrum for 10-methylacridinium iodide **2**.

**Fig. 4:**
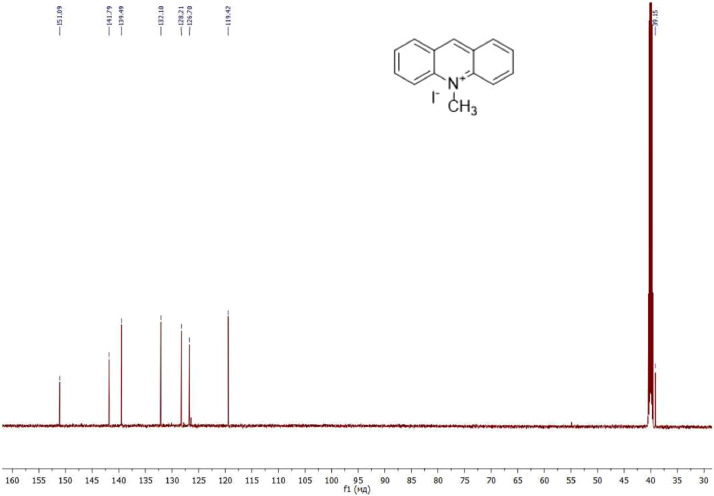
^13^C NMR (100 MHz, DMSO-d6) spectrum for 10-methylacridinium iodide **2**.

**Fig. 5:**
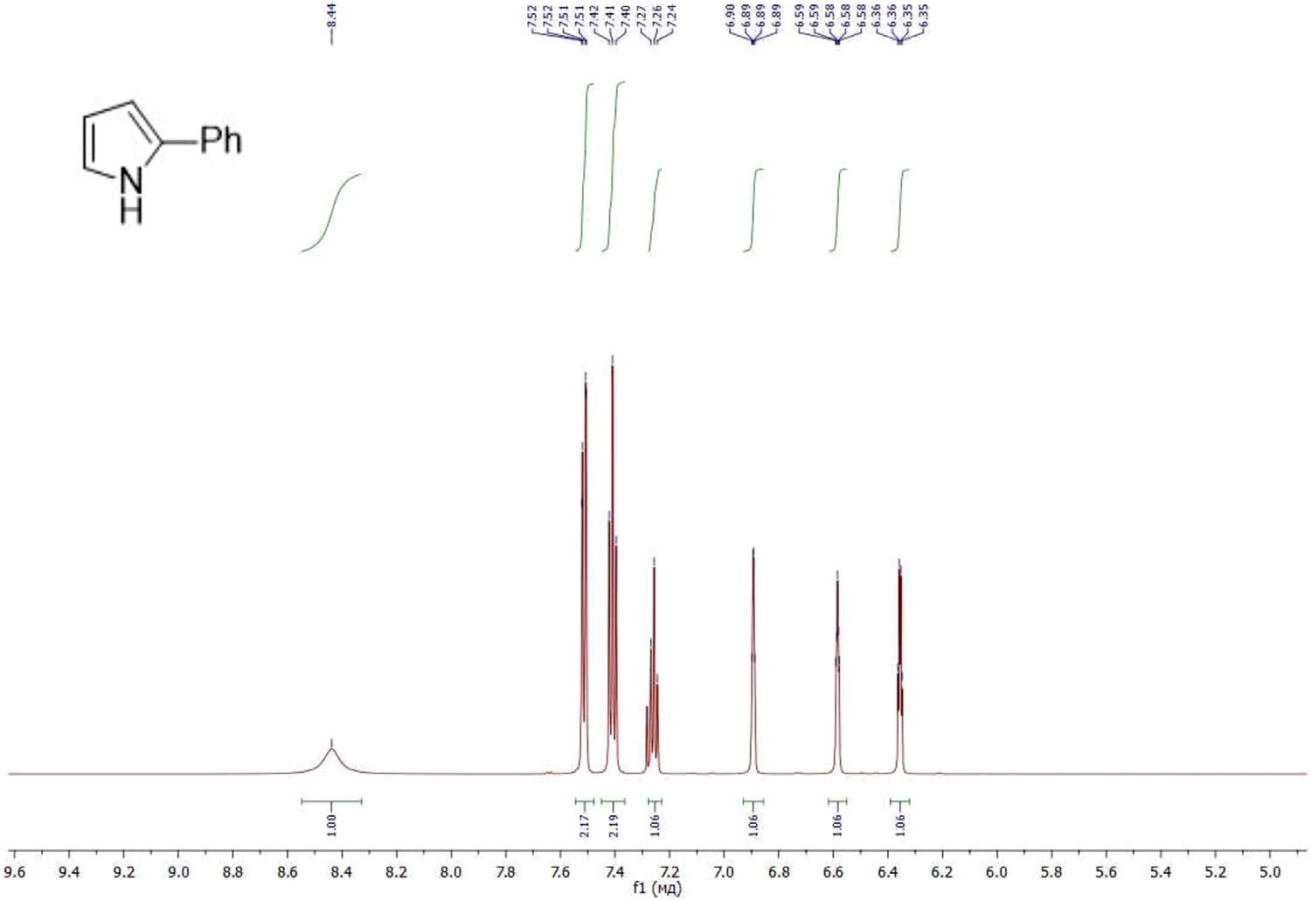
^1^H NMR (400 MHz, CDCl_3_) spectrum for 2-phenyl-1H-pyrrole **3**.

**Fig. 6:**
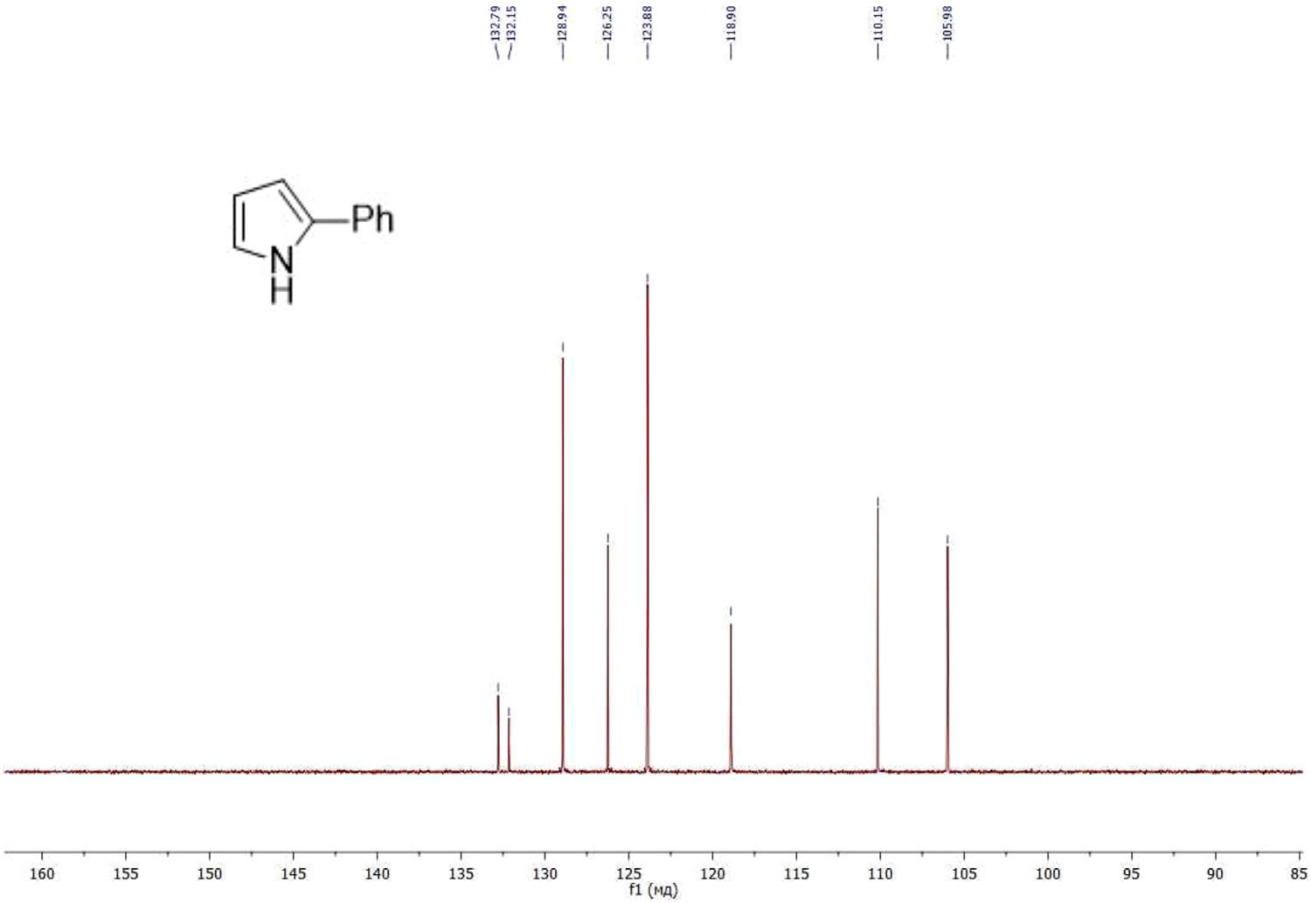
^13^C NMR (100 MHz, CDCl_3_) spectrum for 2-phenyl-1H-pyrrole **3**.

**Fig. 7:**
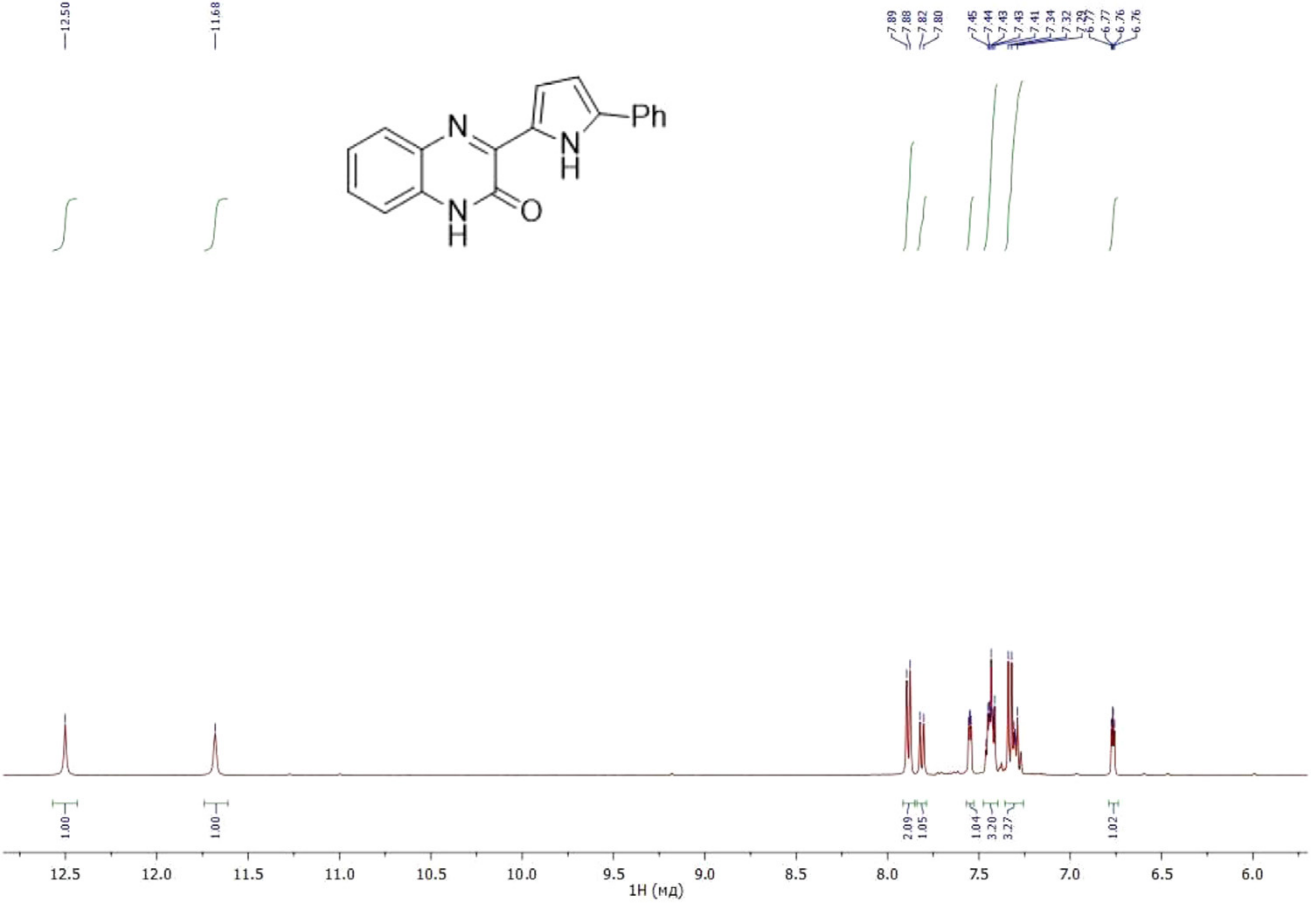
^1^H NMR (400 MHz, DMSO-d6) spectrum for compound **PQ-29**.

**Fig. 8:**
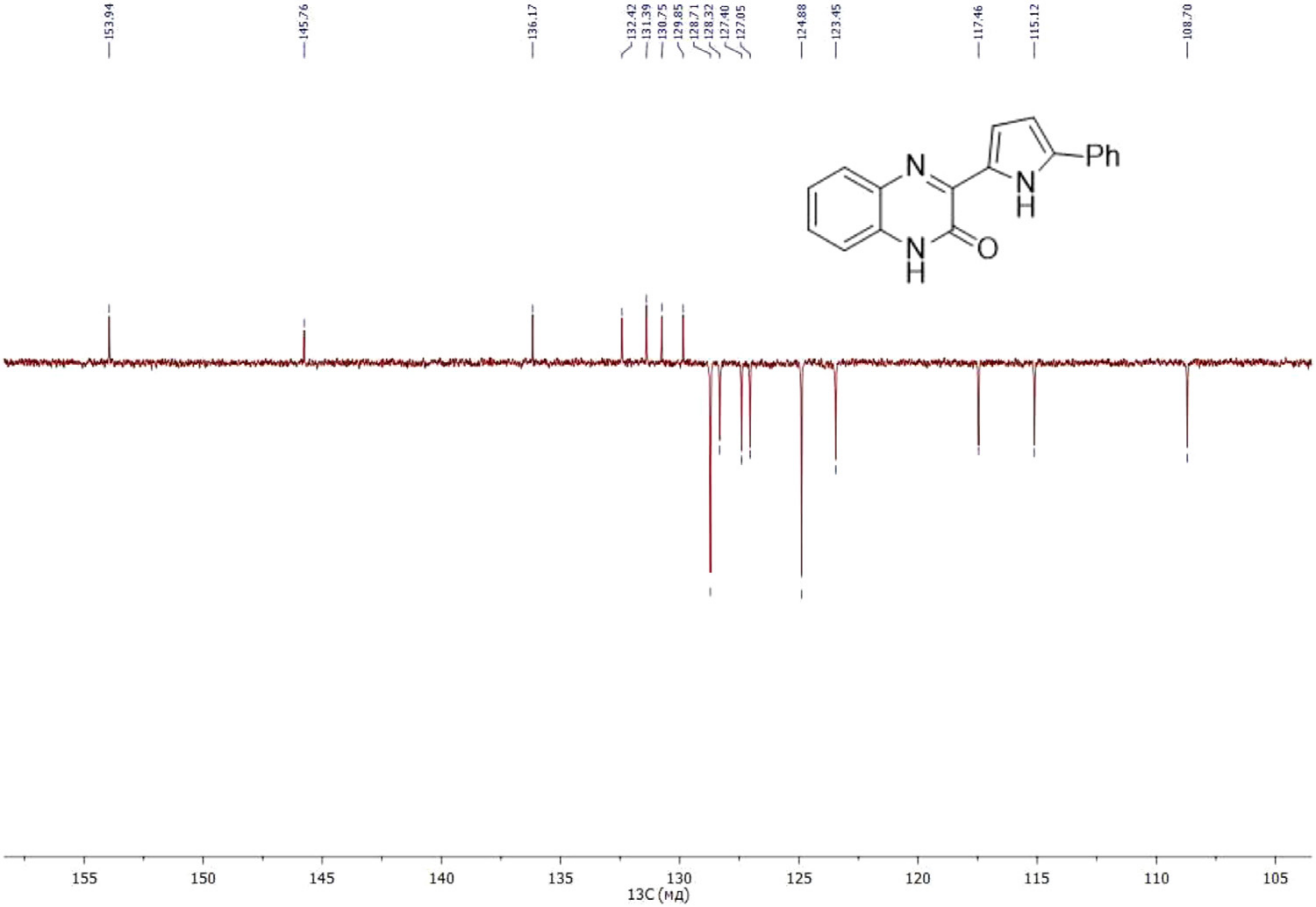
^13^C NMR (100 MHz, DMSO-d6) spectrum for compound **PQ-29**.

**Fig. 9:**
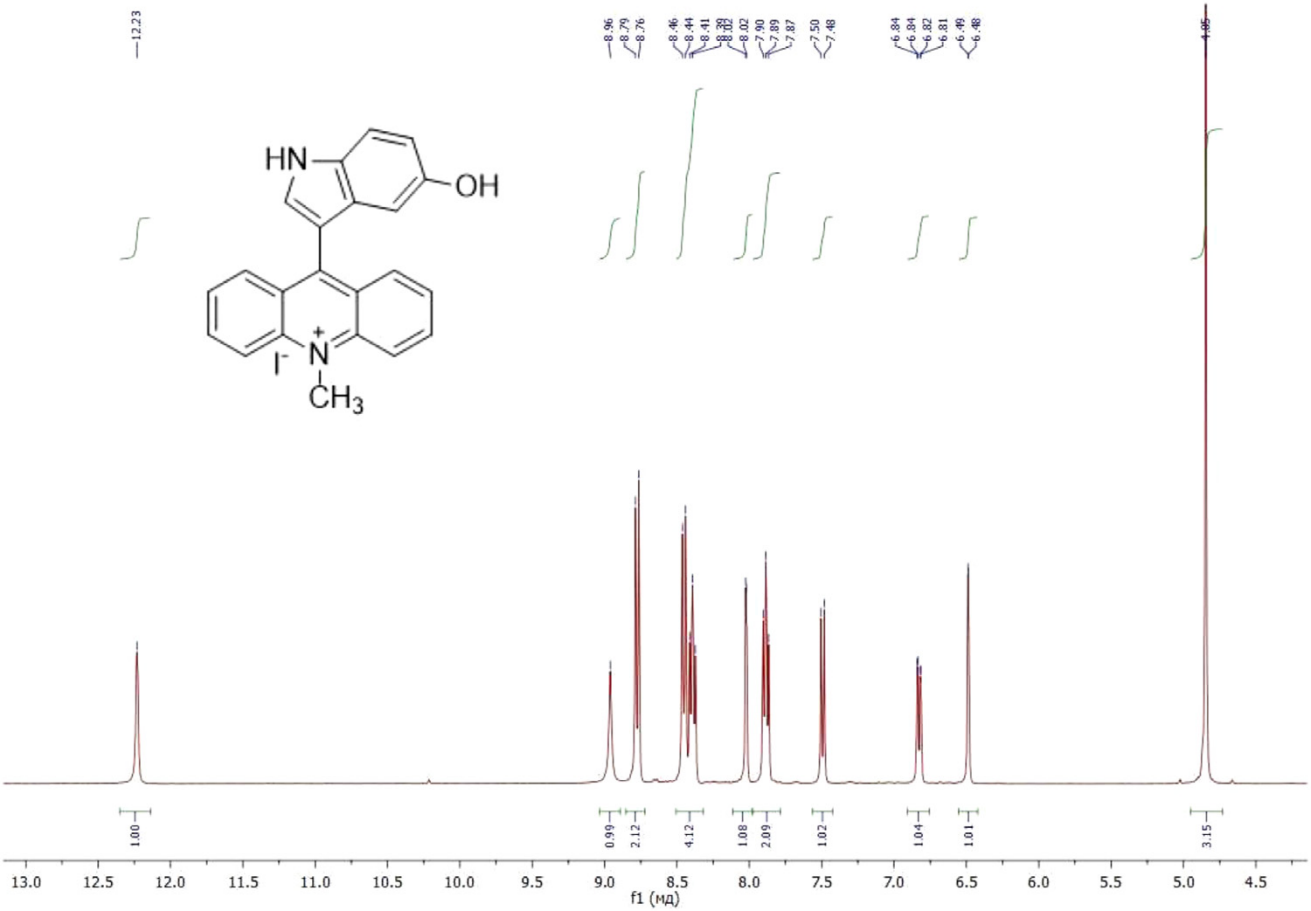
^1^H NMR (400 MHz, DMSO-d6) spectrum for compound **IA-47**.

**Fig. 10:**
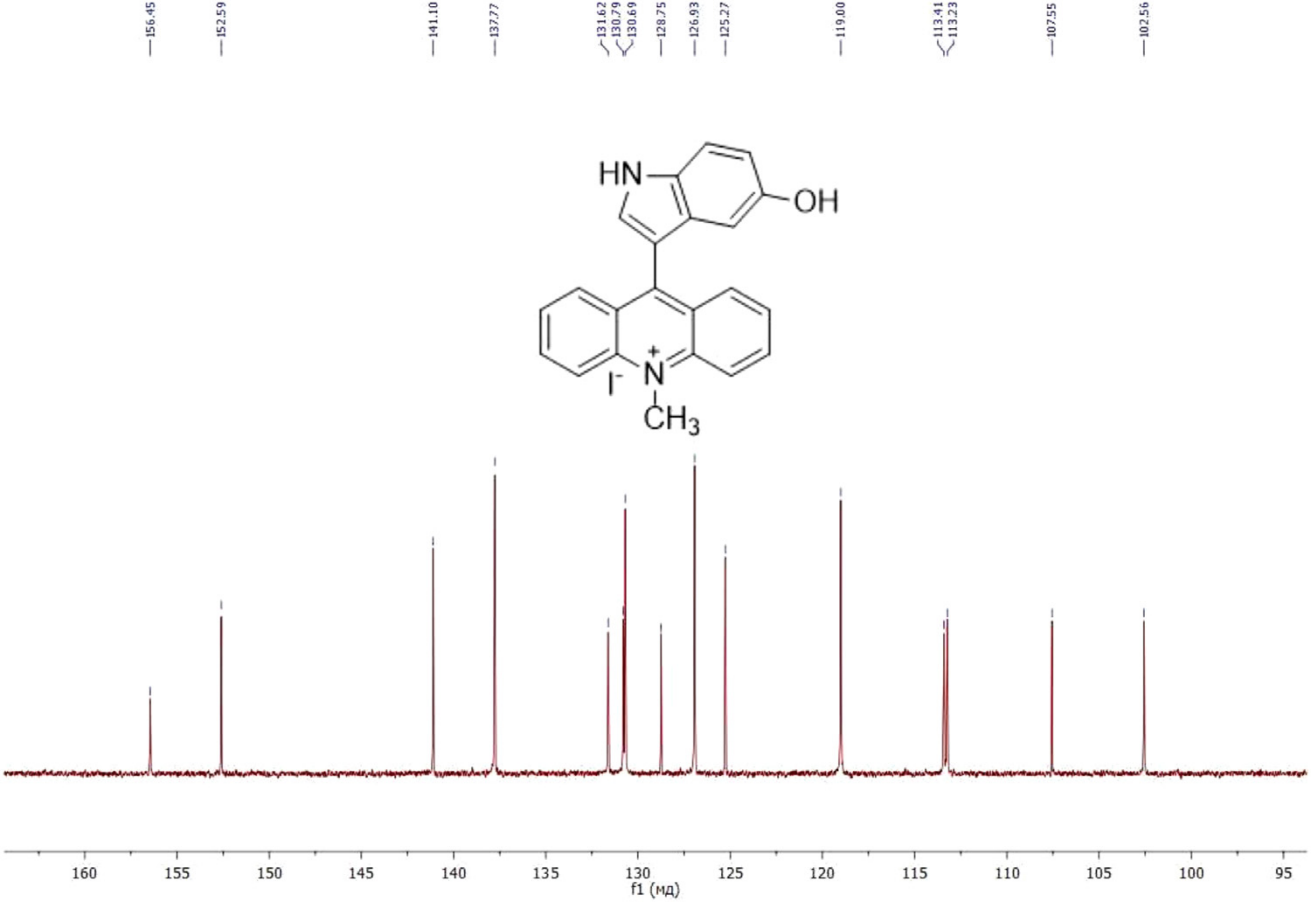
^13^C NMR (100 MHz, DMSO-d6) spectrum for compound **IA-47**.

**Fig. 11:**
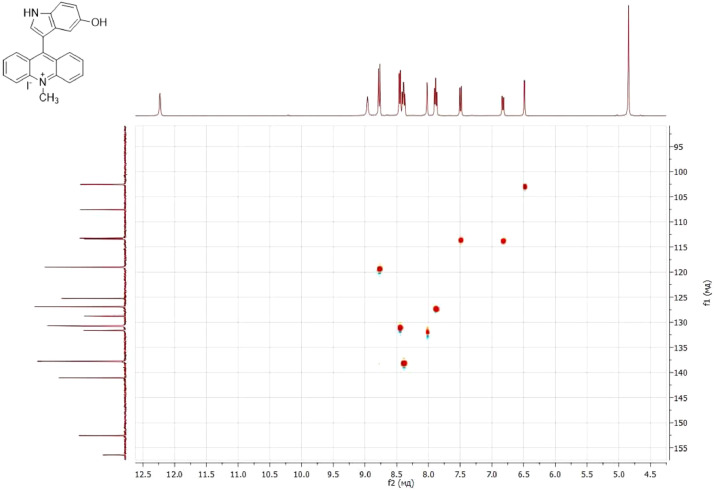
2D ^1^H–^13^C HSQC spectrum for compound **IA-47**.

**Fig. 12:**
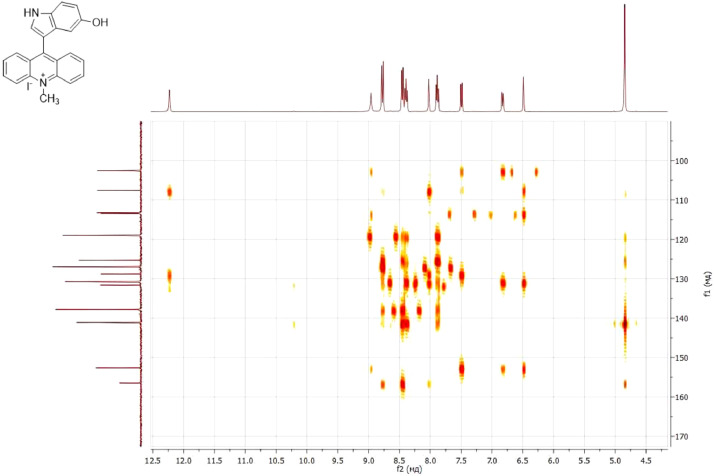
2D ^1^H–^13^C HMBC spectrum for compound **IA-47**.

**Fig. 13:**
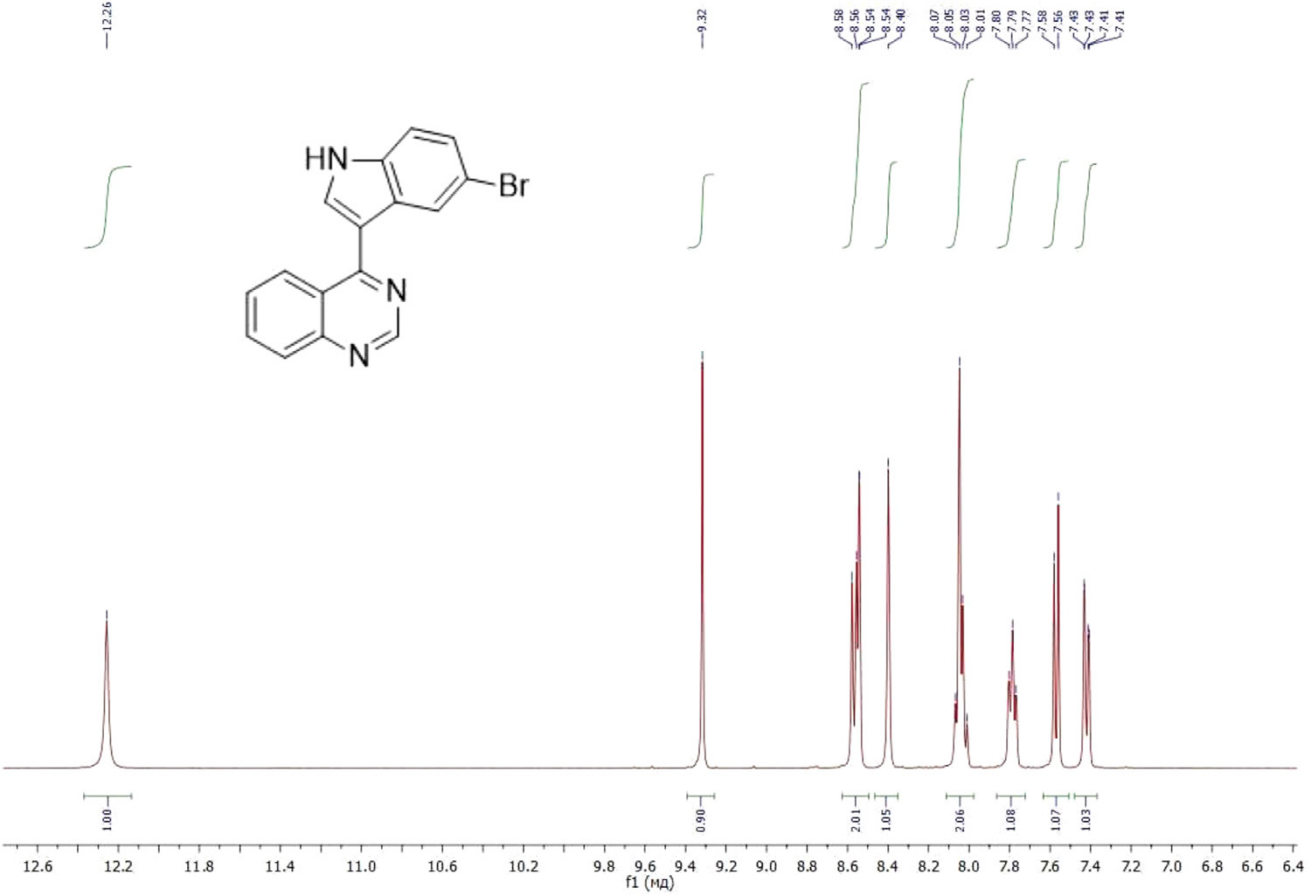
^1^H NMR (400 MHz, DMSO-d6) spectrum for compound **IQ-378**.

**Fig. 14:**
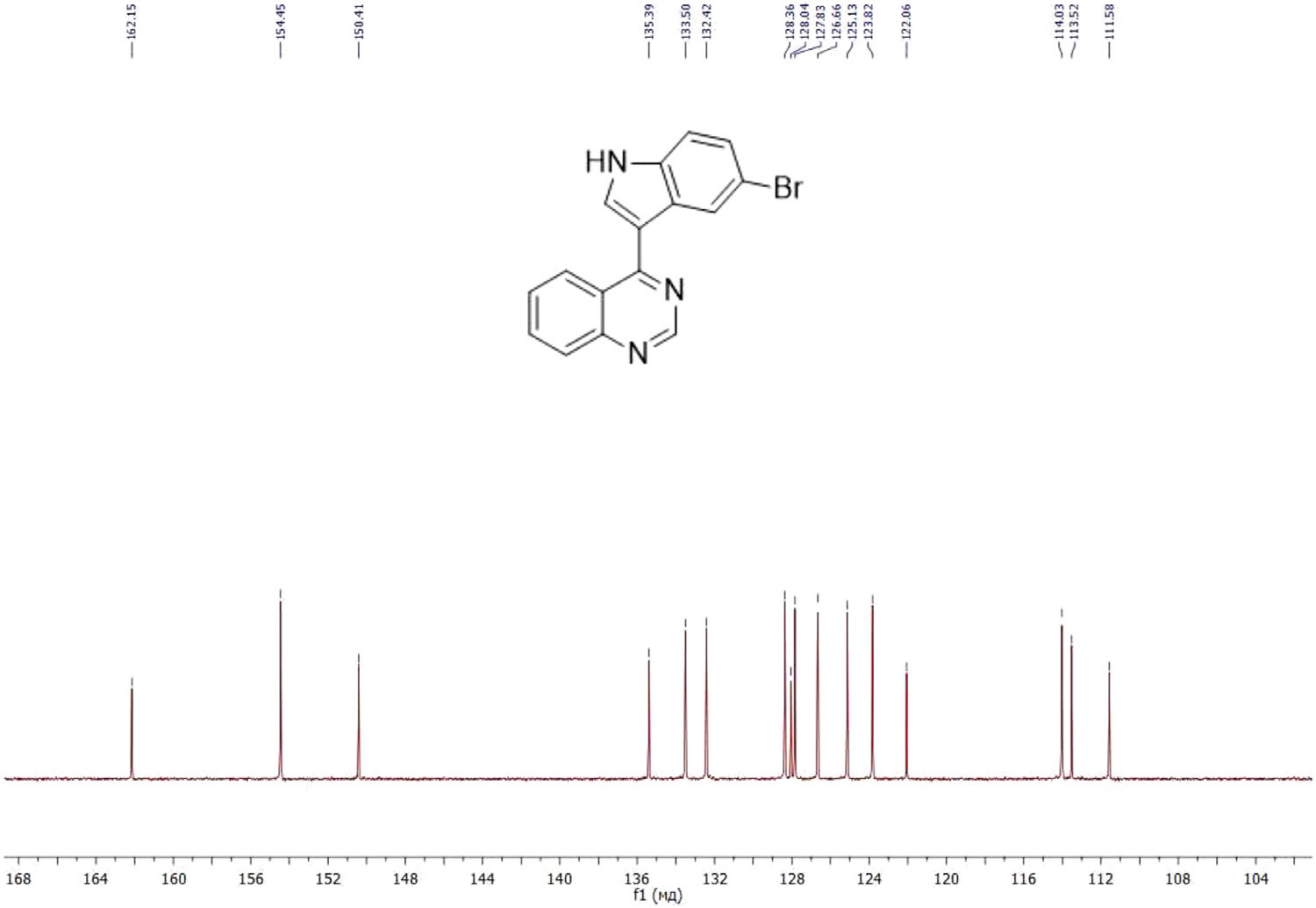
^13^C NMR (100 MHz, DMSO-d6) spectrum for compound **IQ-378**.

**Fig. 15:**
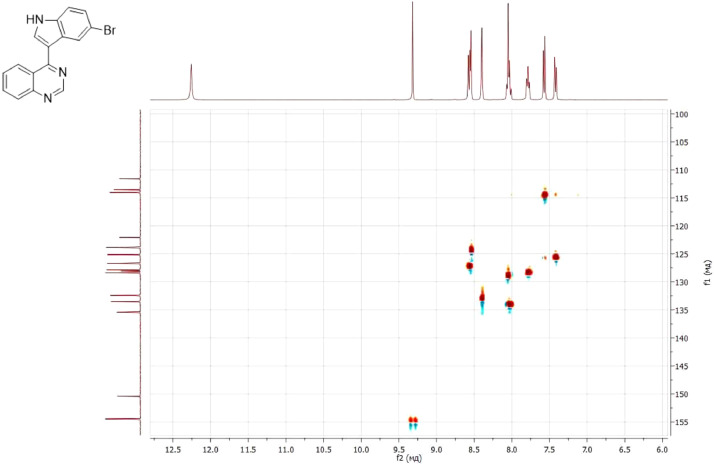
2D ^1^H–^13^C HSQC spectrum for compound **IQ-378**.

**Fig. 16:**
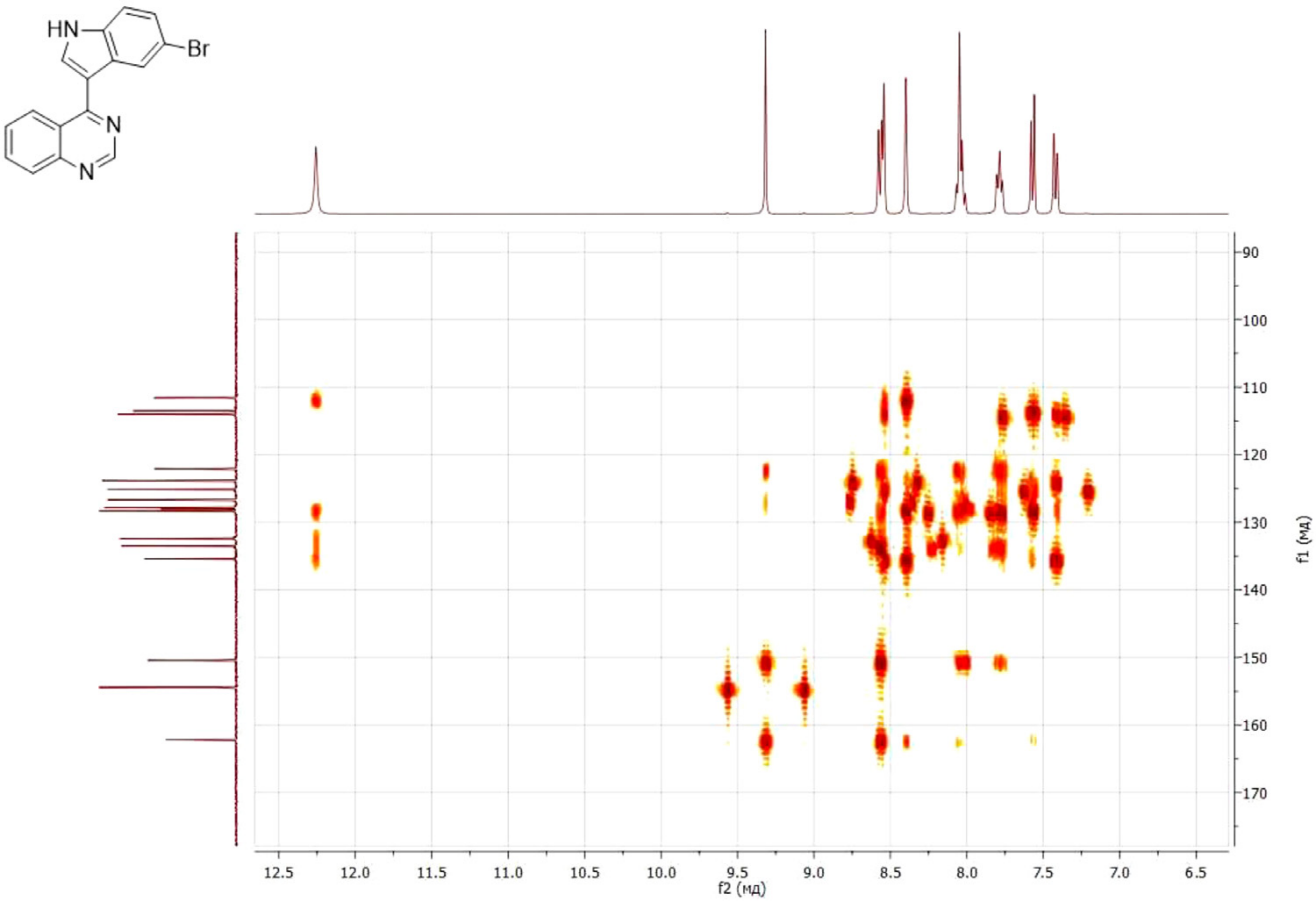
2D ^1^H–^13^C HMBC spectrum for compound **IQ-378**.

**Fig. 17:**
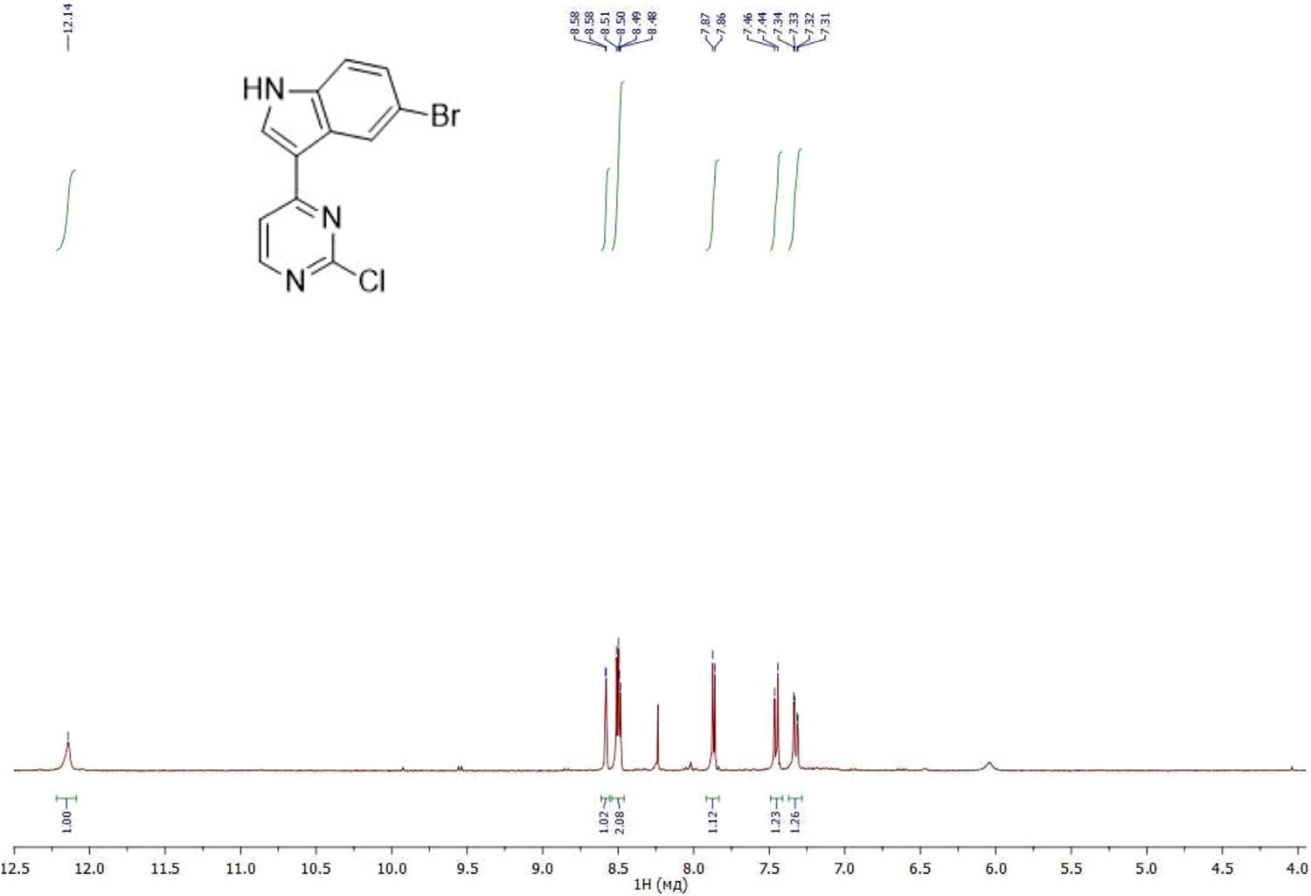
^1^H NMR (400 MHz, DMSO-d6) spectrum for compound **IP-3**.

**Fig. 18:**
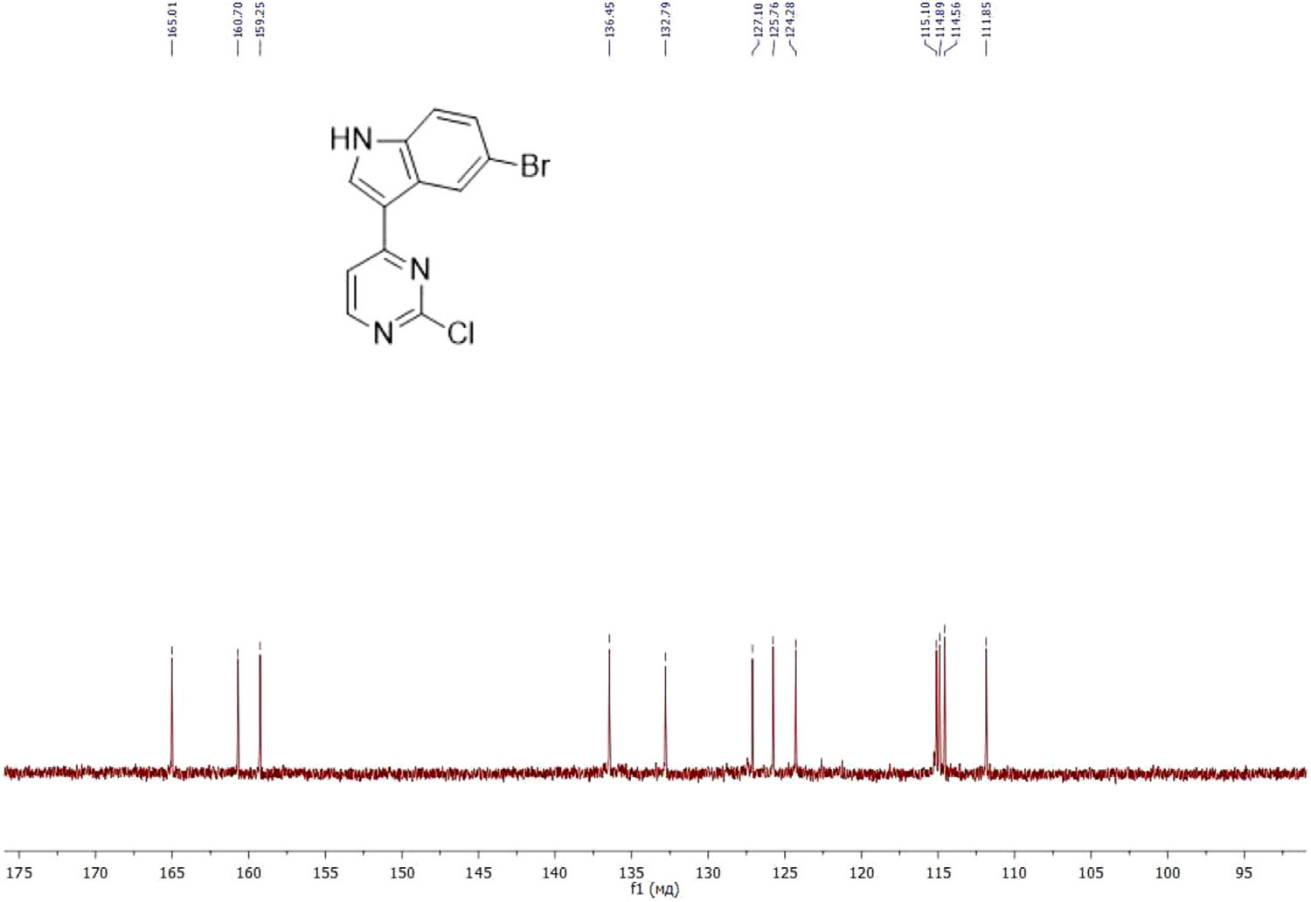
^13^C NMR (100 MHz, DMSO-d6) spectrum for compound **IP-3**.

NMR-spectra for **quinoxalin-2 (1H) -one 1** are presented on Fig. 1, Fig. 2. NMR-spectra for **10-methylacridinium iodide 2** are presented on Figs. 3 and 4. NMR-spectra for **2-phenyl-1H-pyrrole 3** are presented on Figs. 5 and 6. NMR-spectra for **3-(5-phenyl-1*H*-pyrrol-2-yl)quinoxalin-2(1*H*)-one** (**PQ-29**) are presented on Fig. 7 and 8. NMR-spectra for **9-(5-hydroxy-1*H*-indol-3-yl)-10-methylacridinium iodide** (**IA-47**) are presented on Figs. 9–12. NMR-spectra for **4-(5-bromo-1*H*-indol-3-yl)quinazoline** (**IQ-378**) are presented on Figs. 13–16. NMR-spectra for **4-(5-bromo-1*H*-indol-3-yl)-2-chloropyrimidine** (**IP-3**) are presented on Figs. 17 and 18. The row NMR spectra for each substances are presented as a supplemental material.

**Fig. 19:**
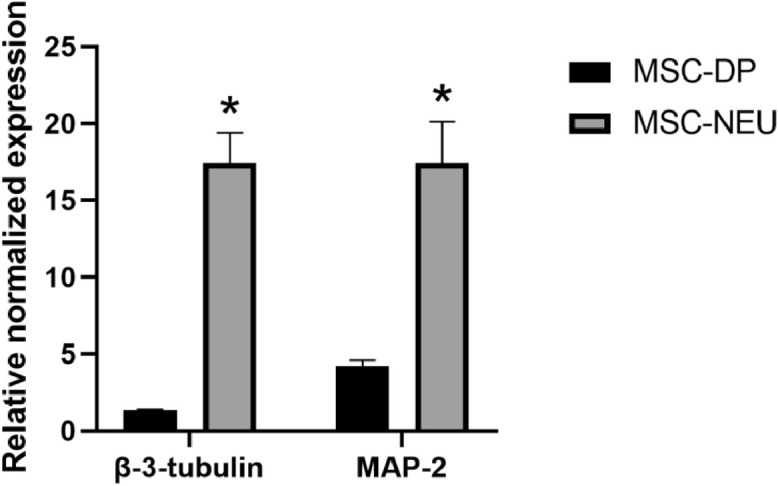
**Verification of neuronal differentiation of MSC cells.** The data of RT-PCR are presented. Histogram bars show the relative amount of mRNA transcribed from the MAP-2 and β-3-tubulin genes gene in MSC-DP or MSC-NEU cells. Data of three independent experiments are presented as mean ± standard error of the mean (SEM). Statistical significance is indicated as * *p* < 0.05.

**Fig. 20:**
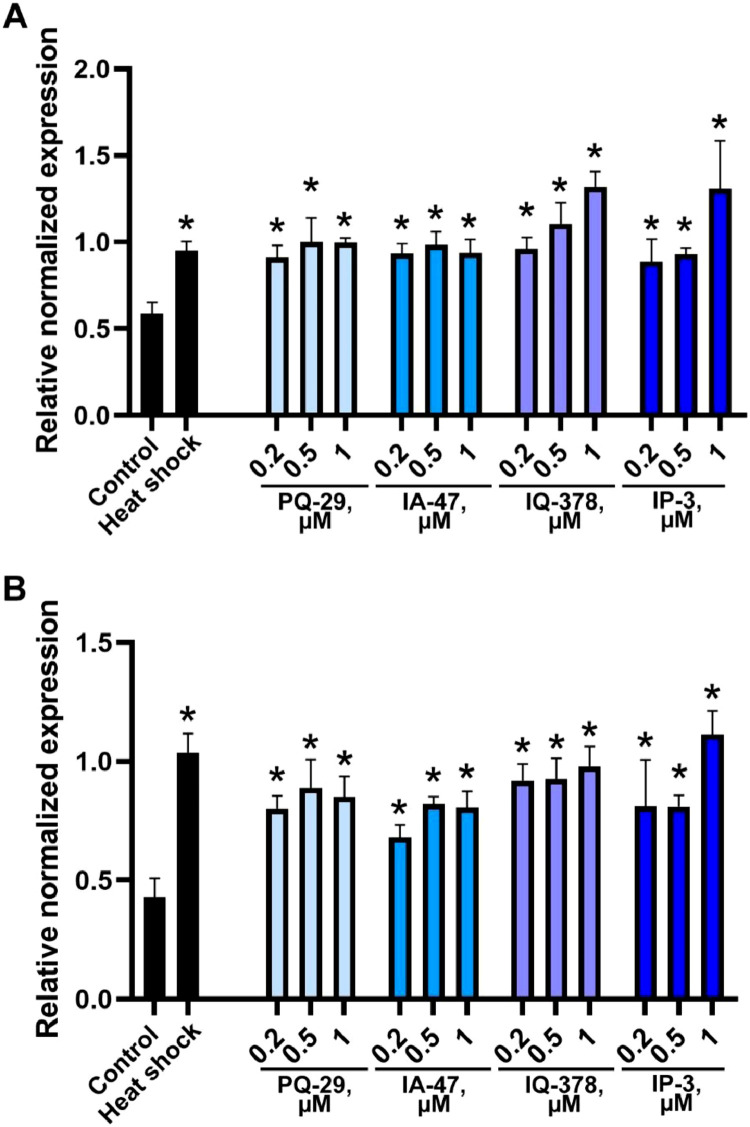
**PLAs and ILAs causes the accumulation of mRNA of heat shock genes in MSC-NEU.** RT-PCR analysis of Hsp70 (A) and Hsp90 (B) mRNA content in lysates of MSC-NEU cells incubated with PLAs and ILAs at the indicated concentrations for 6 h. Data of three independent experiments are presented as mean ± standard error of the mean (SEM). Statistical significance is indicated as * *p* < 0.05.

As we have shown earlier, the neuroprotective effect of the compounds can develop through the induction of the chaperone proteins synthesis. To verify this process, we checked the expression level of HSPA1A and HSP90AA genes in the culture of reprogrammed human neurons. The level of expression of neuronal markers MAP-2 and β-3-tubulin after differentiation of mesenchymal stem cells from human dental pulp is shown in Fig. 19. Fig. 20 shows data of real-time polymerase chain reaction (RT-PCR) demonstrating the effect of pyrrolyl and indolylazines on the expression of the Hsp70 and Hsp90 protein genes. The row results of RT-PCR are presented as a supplemental material.

## 2 Experimental Design, Materials and Methods

^1^H NMR (400 MHz) and ^13^C NMR (100 MHz) spectra were recorded on a Bruker Avance II, using SiMe_4_ as internal reference in DMSO-*d*_6_ and CDCl_3_. Chemical shifts (d) are reported in parts per millions (ppm) and spin multiplicities are given as singlet (s), doublet (d), triplet (t), or multiplet (m). Coupling constants (*J*) are reported in Hz. The mass spectra were recorded on a mass spectrometer SHIMADZU GCMS-QP2010 Ultra with sample ionization by electron impact (EI).

*In vitro* experiments were performed on human mesenchymal stem cells from dental pulp (line MSC-DP), which were isolated, characterized and kindly given to us by Koltsova with co-authors [10]. Cells were cultured in DMEM/F12 medium (Gibco, USA) containing 10% fetal bovine serum (FBS; Gibco, USA), 100 units/mL penicillin, and 0.1 mg/mL streptomycin (BioloT, Russia) at 37 °C and 5% CO_2_. We were reprogramming these cells into a neuronal phenotype for 5 days in Neurobasal medium (Gibco, USA) containing B27 supplement (Gibco, USA), 3% FBS, 100 units/mL penicillin, and 0.1 mg/mL streptomycin.

RNA from cells was isolated using ExtractRNA reagent (Evrogen JSC, Russia) and converted to DNA using a MMLV RT kit (Evrogen JSC, Russia) according to the manufacturer's protocol. All RT-PCR reactions were performed with a CFX96 Real-Time PCR detection system (BioRad, USA) using qPCRmix-HS SYBR (Evrogen JSC, Russia) according to the manufacturer's protocol. Melt curve analysis was employed to prove amplicon accuracy. The data were analyzed for fold-change using Bio-Rad CFX software. The sequence of primers was as follows:

MAP-2: (forward) 5′-CGCTAAATCGTAAGTGAGGGCT-3’, (reverse) 5′-ATTAGAAGTCCCCGC AGTGG-3’;
β-3-tubulin: (forward) 5′-AGCAAGAACAGCAGCTACTTCGT-3’, (reverse) 5′-GATGAAGGTGGA GGACATCTTGA-3’;
GAPDH: (forward) 5′- TGCACCACCAACTGCTTAGC-3’, (reverse) 5′-GGCATGGACTGTGGTCATGAG-3’;
Hsp70: (forward) 5′-AGAAGGACATCAGCCAGAACAA-3’, (reverse) 5′-AGAAGTCGATGCCCTC AAACA-3’;
Hsp90: (forward) 5′-ACTCTTTACTGAACTGGCGGAA-3’, (reverse) 5′-AGAGTCTTCGTGTA TTCCAAGCT-3’.

GAPDH was used as the normalization control. All primers were obtained from Evrogen JSC (Russia). The parameters of the PCR were 5 min of pre-denaturation at 95 °C, followed by 40 cycles of 30 s at 95 °C, 30 s at 65 °C, and 30 s at 70 °C. The data were analyzed for fold change (ΔΔCt) using BioRad CFX software (version 3.1).

## CRediT authorship contribution statement

**Elizaveta A. Dutysheva:** Investigation. **Irina A. Utepova:** Writing – review & editing, Funding acquisition. **Maria A. Trestsova:** Visualization, Investigation. **Alexander S. Anisimov:** Software. **Valery N. Charushin:** Project administration. **Oleg N. Chupakhin:** Supervision. **Boris A. Margulis:** Supervision, Writing – review & editing. **Irina V. Guzhova:** Conceptualization, Writing – review & editing. **Vladimir F. Lazarev:** Data curation, Writing – original draft, Funding acquisition.

## Declaration of Competing Interest

The authors declare that they have no known competing financial interests or personal relationships which have or could be perceived to have influenced the work reported in this article.

## Appendix Supplementary materials

Image, application 1
